# Time‐trends and age and stage differences in 5‐year relative survival for common cancer types by sex in the canton of Zurich, Switzerland

**DOI:** 10.1002/cam4.6392

**Published:** 2023-08-01

**Authors:** Miriam Wanner, Maria‐Eleni Syleouni, Nena Karavasiloglou, Manuela Limam, Esther Bastiaannet, Dimitri Korol, Sabine Rohrmann

**Affiliations:** ^1^ Cancer Registry Zurich, Zug, Schaffhausen and Schwyz, Institute of Pathology and Molecular Pathology University Hospital Zurich Zurich Switzerland; ^2^ Epidemiology, Biostatistics and Prevention Institute University of Zurich Zurich Switzerland; ^3^ European Food Safety Authority Parma Italy

**Keywords:** cancer registry, relative survival, time trends

## Abstract

**Background:**

Survival trends help to evaluate the progress made to reduce the burden of cancer. The aim was to estimate the trends in 5‐year relative survival of patients diagnosed with breast, prostate, lung, colorectal cancer and skin melanoma in the time periods 1980–1989, 1990–1999, 2000–2009 and 2010–2015 in the Canton of Zurich, Switzerland. Furthermore, we investigated relative survival differences by TNM stage and age group.

**Methods:**

Data from the Cancer Registry of Zurich was used from 1980 to and including 2015, including incident cases of breast (*N* = 26,060), prostate (*N*= 23,858), colorectal (*N*= 19,305), lung cancer (*N*= 16,858) and skin melanoma (*N*= 9780) with follow‐up until 31 December 2020. The cohort approach was used to estimate 5‐year relative survival.

**Results:**

The 5‐year relative survival increased significantly between 1980 and 1989, and 2010 and2015: from 0.70 to 0.89 for breast, from 0.60 to 0.92 for prostate, from 0.09 to 0.23 (men) and from 0.10 to 0.27 (women) for lung, from 0.46 to 0.66 (men) and from 0.48 to 0.68 (women) for colorectal cancer, and from 0.74 to 0.94 (men) and from 0.86 to 0.96 (women) for skin melanoma. Survival for stage IV tumors was considerably lower compared to lower‐staged tumors for all cancer types. Furthermore, relative survival was similar for the age groups <80 years but lower for patients aged 80 years and older.

**Conclusion:**

The observed increasing trends in survival are encouraging and likely reflect raised awareness around cancer, improved diagnostic methods, and improved treatments. The fact that stage I tumor patients have generally high relative survival reflects the efforts made regarding early detection.

## Graphical abstract

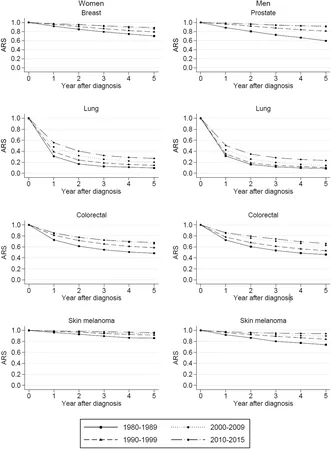

## 1 INTRODUCTION

Cancer diagnosis and treatment have changed greatly during the last decades. In combination with trends in incidence and mortality, survival trends help to evaluate the progress made against cancer.^1^ In Switzerland as well as in the canton of Zurich (the largest Swiss canton), the most common types of cancer are breast (women) and prostate (men), lung, colorectal cancer and skin melanoma.^2^ In general, breast, prostate cancer and skin melanoma incidence have been increasing in the canton of Zurich since 1980.^3^ Regarding lung cancer incidence, an increase was only observed in women, while for men the trend was decreasing.^3^ The trend for colorectal cancer was stable in both sexes.^3^ In the same period, stable or decreasing mortality rates were observed for these cancers, except for lung cancer in women.^3^ This is also seen in other countries.^3^, ^4^, ^5^, ^6^

Large international studies such as EUROCARE^7^, ^8^ and CONCORD^9^ have provided survival estimates, indicating better survival probability in Central and Northern Europe compared to Eastern Europe.^7^ Cancer survival seems to increase in all regions, with higher increases in regions that had previously lower survival probabilities.^7^

In the cancer registry of the canton of Zurich, an active follow‐up is performed based on data from the resident control that dates back to 2012 (including relocations within and away from the canton and information regarding the date of death) and on the Central Compensation Office that dates back to 2005 (including information regarding the date of death). In earlier years, the registry performed a passive follow‐up with anonymous linkage to the causes of death statistics. The high percentage of available information regarding the vital status of the patients enabled us to conduct survival analyses for patients diagnosed in the canton of Zurich.

The aim of the present study was to estimate time trends in age‐standardized 5‐year relative survival of patients diagnosed with one of the most frequently diagnosed types of cancer (i.e., breast, prostate, lung, colorectal, skin melanoma) between 1980 and 2015 by sex. Furthermore, we investigated relative survival differences by TNM stage and age group.

## 2 MATERIALS AND METHODS

### 2.1 Data

The cancer registry of the canton of Zurich began registering data in 1980. It covers a population of between 1.1 million and 1.5 million inhabitants in 1980 and 2020, respectively.^10^ Three other cantons have since joined the registry: in 2011 (Zug) and in 2020 (Schaffhausen and Schwyz); however, these data are not included in the present analyses due to the short follow‐up period. The patient's main place of residence at the time of diagnosis is the criterion for inclusion in the respective cantonal cancer registry. In general, the data quality in the cancer registry of the canton of Zurich is good.^11^ The percentage of death certificate only (DCO) cases was 2.6% and the percentage of morphologically verified cases was 93.3% for the period 1997–2014.^11^

We included patients with primary malignant tumors diagnosed in the canton of Zurich between 1980 and 2015 according to the following International Statistical Classification of Diseases and Related Health Problems 10th Revision (ICD‐10) codes: breast (C50), prostate (C61), colorectal (C18–C21), lung (C33–C34), and skin melanoma (C43). We excluded DCO cases. Furthermore, if a person had more than one malignant cancer diagnosis, we only included the first incident case. If there were two different diagnoses on the same date, the patient was excluded.

In order to calculate 5‐year relative survival using the cohort approach,^12^ we included incidence data up to and including 2015 with follow‐up until December 31, 2020.

The dataset includes information on the date of diagnosis, the ICD‐10 code, age at diagnosis, clinical and pathological TNM to define stage, basis of diagnosis (indication of DCO), vital status at follow‐up, and date of follow‐up. Patients' survival was assessed through December 31, 2020. The vital status of patients with a follow‐up date before December 31, 2020 was set to lost‐to‐follow‐up using the date of last contact as follow‐up date.

In order to investigate time trends, we used four time periods: 1980–1989, 1990–1999, 2000–2009, and 2010–2015 (cohort analyses). Due to the large amount of missing data for stage in earlier years, we only used data from patients diagnosed between 2003 and 2015 in the stage‐stratified analyses. TNM stage was defined as stage I, stage II, stage III, stage IV and missing stage according to the TNM classification of malignant tumors version 6 and 7.^13^, ^14^ For age‐stratified analyses, we categorized the individuals into the age groups <60 years, 60–69 years, 70–79 years, and ≥ 80 years based on their age at diagnosis; for breast cancer and skin melanoma, we used an additional younger age group (<50, 50–59, etc.). We then calculated 5‐year relative survival separately for each age group.

### 2.2 Ethics

In the Canton of Zurich, all cancer cases are registered with presumed consent and registered based on a decision from 1980 by the Zurich Government Council and the general registry approval from 1995 by the Federal Commission of Experts for professional secrecy in medical research. In this analysis, all data were used anonymously, and therefore no approval was required from the Ethics Committee of the Canton of Zurich.

### 2.3 Statistical analyses

We estimated age‐standardized 5‐year relative survival as the ratio of the observed survival to the expected survival without taking cause of death into account. Expected survival was calculated based on the Ederer II method^15^ applied to all‐cause mortality tables for the canton of Zurich supplied by the Federal Statistical Office. Death probabilities based on age‐, sex‐, and calendar year‐specific death rates were interpolated and smoothed using the Elandt‐Johnson formula.^16^ Relative survival was developed as an estimator of net survival, which is commonly used when estimating patient survival using data from population‐based cancer registries.^12^

Relative survival was estimated using the strs command in Stata Statistical Software (StataCorp LP, version 15). Cohort analyses were used to derive relative survival estimates for all time periods (2010–2015 for the latest period in order to provide at least 5 years of follow‐up). All analyses were performed using Stata Statistical software (SE version 15).

## 3 RESULTS

Table 1 shows the number of cases by cancer type, sex, time period, age at diagnosis and stage at diagnosis. Overall, 26,060 women with breast cancer, 23,858 men with prostate cancer, 16,858 individuals with lung cancer, 19,305 individuals with colorectal cancer, and 9780 individuals with skin melanoma were included in the analyses.

**TABLE 1:** Number of patients for each cancer type and sex, by period of diagnosis (1980–2015), age at diagnosis (1980–2015) and stage at diagnosis (2003–2015). Canton of Zurich, Switzerland.

	Breast cancer	Prostate cancer	Lung cancer		Colorectal cancer		Skin melanoma	
	Women *N* (%)	Men *N* (%)	Men *N* (%)	Women *N* (%)	Men *N* (%)	Women *N* (%)	Men *N* (%)	Women *N* (%)
Overall (1980–2015)	26,060 (100.0)	23,858 (100.0)	11,807 (100.0)	5051 (100.0)	9948 (100.0)	9357 (100.0)	4758 (100.0)	5022 (100.0)
Diagnosis period								
1980–1989	5343 (20.5)	3536 (14.8)	3535 (29.9)	768 (15.2)	2339 (23.5)	2348 (25.1)	690 (14.5)	935 (18.6)
1990–1999	6536 (25.1)	5728 (24.0)	3196 (27.1)	1144 (22.6)	2501 (25.1)	2450 (26.2)	1087 (22.8)	1162 (23.1)
2000–2009	8520 (32.7)	9122 (38.2)	3156 (26.7)	1770 (35.0)	3158 (31.7)	2839 (30.3)	1586 (33.3)	1558 (31.0)
2010–2015	5661 (21.7)	5472 (22.9)	1920 (16.3)	1369 (27.1)	1950 (19.6)	1720 (18.4)	1395 (29.3)	1367 (27.2)
Age at diagnosis								
<50 years^b^	5918 (22.7)						1225 (25.7)	1871 (37.3)
<60^c^/50–59 years	5706 (21.9)	2817 (11.8)	2940 (24.9)	1474 (29.2)	2296 (23.1)	1997 (21.3)	846 (17.8)	905 (18.0)
60–69 years	6253 (24.0)	8416 (35.3)	3878 (32.8)	1484 (29.4)	2783 (28.0)	2070 (22.1)	1126 (23.7)	889 (17.7)
70–79 years	4977 (19.1)	8460 (35.5)	3556 (30.1)	1389 (27.5)	3055 (30.7)	2800 (29.9)	1035 (21.8)	762 (15.2)
80+ years	3206 (12.3)	4165 (17.5)	1433 (12.1)	704 (13.9)	1814 (18.2)	2490 (26.6)	526 (11.1)	595 (11.8)
Overall (2003–2015)^a^	11,725 (100.0)	12,083 (100.0)	4165 (100.0)	2720 (100.0)	4145 (100.0)	3738 (100.0)	2616 (100.0)	2557 (100.0)
Stage at diagnosis								
Stage I	4176 (35.6)	2521 (20.9)	477 (11.5)	369 (13.6)	723 (17.4)	609 (16.3)	1193 (45.6)	1210 (47.3)
Stage II	4662 (39.8)	4530 (37.5)	287 (6.9)	141 (5.2)	1031 (24.9)	918 (24.6)	130 (5.0)	107 (4.2)
Stage III	1858 (15.8)	1202 (9.9)	720 (17.3)	476 (17.5)	1147 (27.7)	1019 (27.3)	118 (4.5)	81 (3.2)
Stage IV	708 (6.0)	1280 (10.6)	1666 (40.0)	1123 (41.3)	812 (19.6)	672 (18.0)	87 (3.3)	45 (1.8)
Missing stage	321 (2.7)	2550 (21.1)	1015 (24.4)	611 (22.5)	432 (10.4)	520 (13.9)	1088 (41.6)	1114 (43.6)

^a^ Diagnosis period for analyses stratified by stage at diagnosis.

^b^ Youngest age group for breast cancer and skin melanoma.

^c^ Youngest age group for prostate, lung and colorectal cancer.

### 3.1 Age‐standardized 5‐year relative survival by period

**FIGURE 1:**
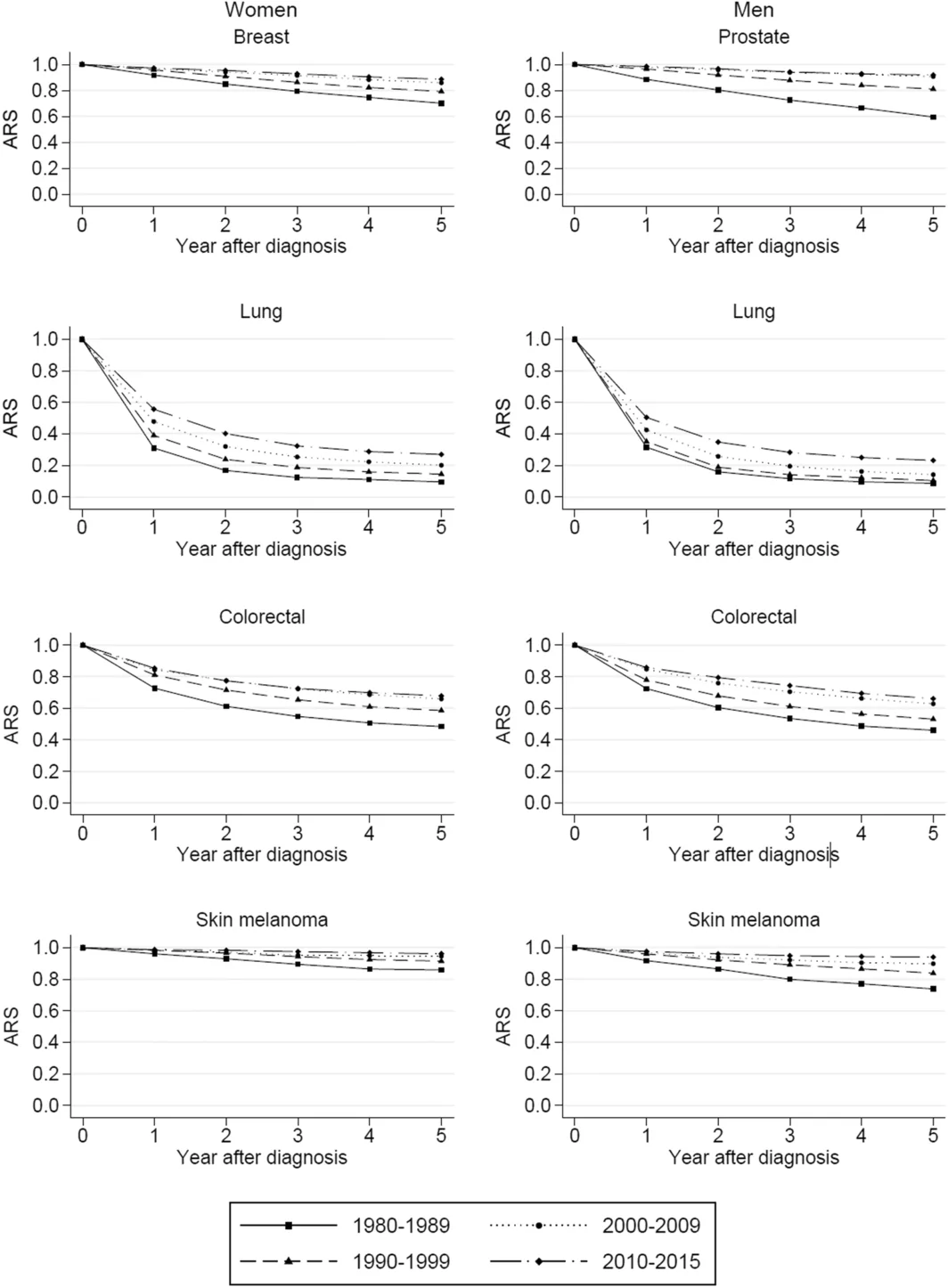
Age‐standardized relative survival (ARS) up to 5 years after diagnosis for each cancer type and sex, by period of diagnosis. Canton of Zurich, Switzerland, 1980–2015. Cohort analyses.

Figure 1 and Table 2 present the age‐standardized 5‐year relative survival estimates by cancer type, sex and time period. In general, the age‐standardized 5‐year relative survival improved over time from the time period 1980–1989 to the time period 2010–2015 for all cancer types and both sexes and was generally slightly higher in women compared to men.

**TABLE 2:** Age‐standardized 5‐year relative survival (ARS) and 95% confidence intervals (95% CI) for each cancer type and period of diagnosis by sex. Canton of Zurich, Switzerland, 1980–2015. Cohort analyses.

Cancer type	5‐year ARS	95% CI	5‐year ARS	95% CI
	Men		Women	
Breast cancer				
Overall (1980–2015)			0.82	(0.81; 0.82)
1980–1989			0.70	(0.68; 0.72)
1990–1999			0.79	(0.78; 0.81)
2000–2009			0.86	(0.85; 0.87)
2010–2015			0.89	(0.87; 0.90)
Prostate cancer				
Overall (1980–2015)	0.85	(0.84; 0.86)		
1980–1989	0.60	(0.56; 0.63)		
1990–1999	0.81	(0.79; 0.83)		
2000–2009	0.91	(0.90; 0.92)		
2010–2015	0.92	(0.91; 0.93)		
Lung cancer				
Overall (1980–2015)	0.13	(0.12; 0.14)	0.19	(0.18; 0.20)
1980–1989	0.09	(0.08; 0.10)	0.10	(0.08; 0.12)
1990–1999	0.10	(0.09; 0.12)	0.14	(0.12; 0.17)
2000–2009	0.14	(0.13; 0.16)	0.20	(0.18; 0.22)
2010–2015	0.23	(0.21; 0.25)	0.27	(0.24; 0.30)
Colorectal cancer				
Overall (1980–2015)	0.57	(0.56; 0.58)	0.60	(0.59; 0.61)
1980–1989	0.46	(0.44; 0.48)	0.48	(0.46; 0.51)
1990–1999	0.53	(0.51; 0.55)	0.59	(0.56; 0.61)
2000–2009	0.63	(0.61; 0.65)	0.66	(0.64; 0.68)
2010–2015	0.66	(0.64; 0.68)	0.68	(0.65; 0.70)
Skin melanoma				
Overall (1980–2015)	0.86	(0.84; 0.88)	0.91	(0.89; 0.93)
1980–1989	0.74	(0.70; 0.78)	0.86	(0.83; 0.89)
1990–1999	0.84	(0.81; 0.86)	0.92	(0.89; 0.93)
2000–2009	0.90	(0.88; 0.92)	0.95	(0.93; 0.96)
2010–2015	0.94	(0.92; 0.96)	0.96	(0.94; 0.97)

For example, the age‐standardized 5‐year relative survival for breast cancer was 0.82 (95% confidence intervals [95% CI] 0.81, 0.82) overall and improved from 0.70 (0.68, 0.72) for the period 1980–1989 to 0.89 (0.87, 0.90) for the period 2010–2015. For lung cancer, overall survival was higher for women than for men but improved over time for both sexes.

### 3.2 Age‐standardized 5‐year relative survival by stage at diagnosis

**FIGURE 2:**
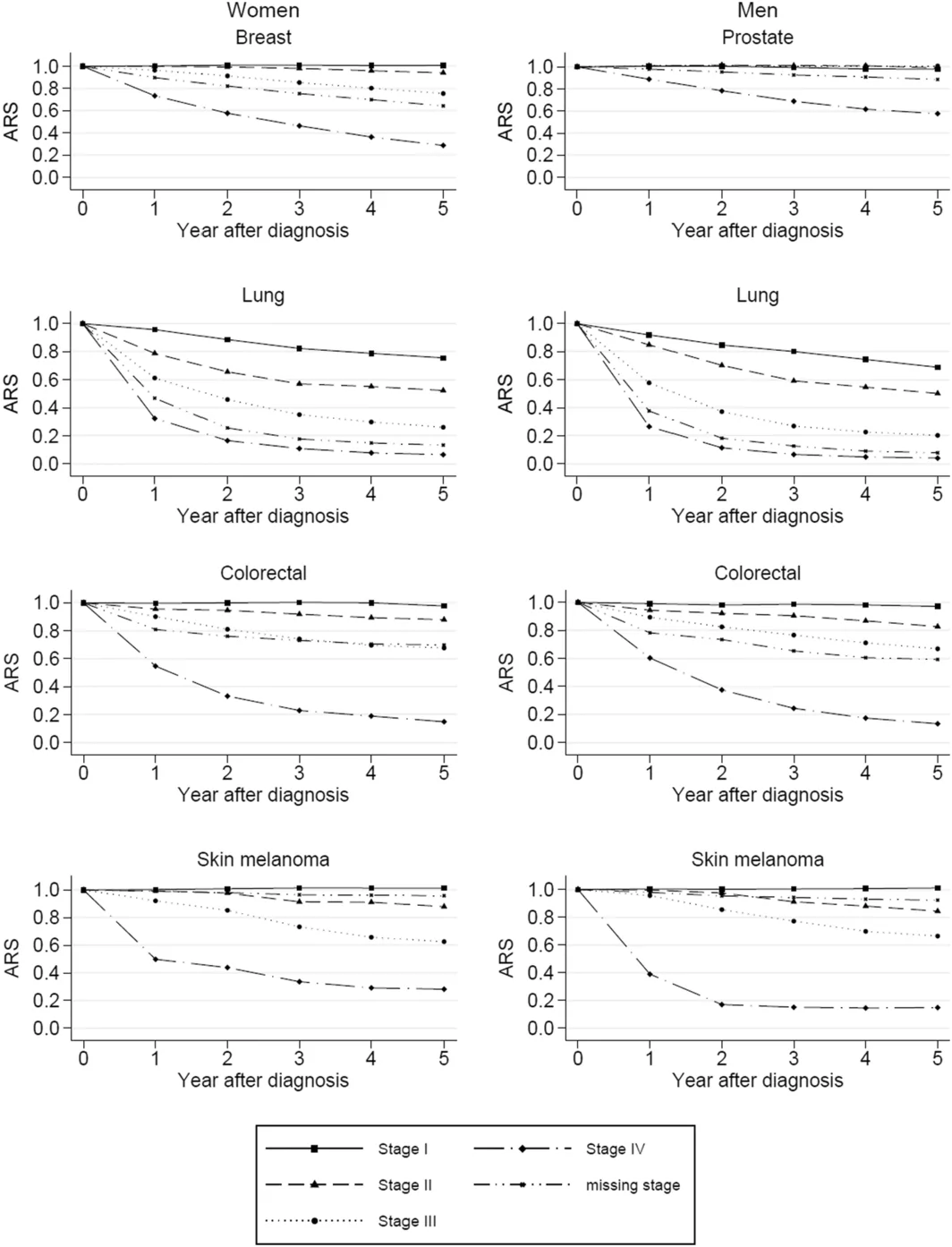
Age‐standardized relative survival (ARS) up to 5 years after diagnosis for each cancer type and sex, by TNM stage. Canton of Zurich, Switzerland, 2003–2015. Cohort analyses.

Figure 2 and Table S1 show the age‐standardized 5‐year relative survival estimates by cancer type, sex, and stage at diagnosis. In general, the age‐standardized 5‐year relative survival was lowest in patients with stage IV tumors for all cancer types. While for most cancer types (breast, lung, colorectal cancer, skin melanoma), 5‐year relative survival decreased with increasing stage, this was not the case for prostate cancer patients, where 5‐year relative survival was similarly high for patients with stage I, II and III tumors but only clearly lower in patients with stage IV tumors.

For all cancer types except for lung cancer, 5‐year relative survival for stage I tumors was equal or even slightly higher compared to the general population. Regarding stage IV tumors, 5‐year relative survival was low for all cancer types (below 0.30 except for prostate cancer where it was 0.58).

### 3.3 5‐year relative survival by age group at diagnosis

**FIGURE 3:**
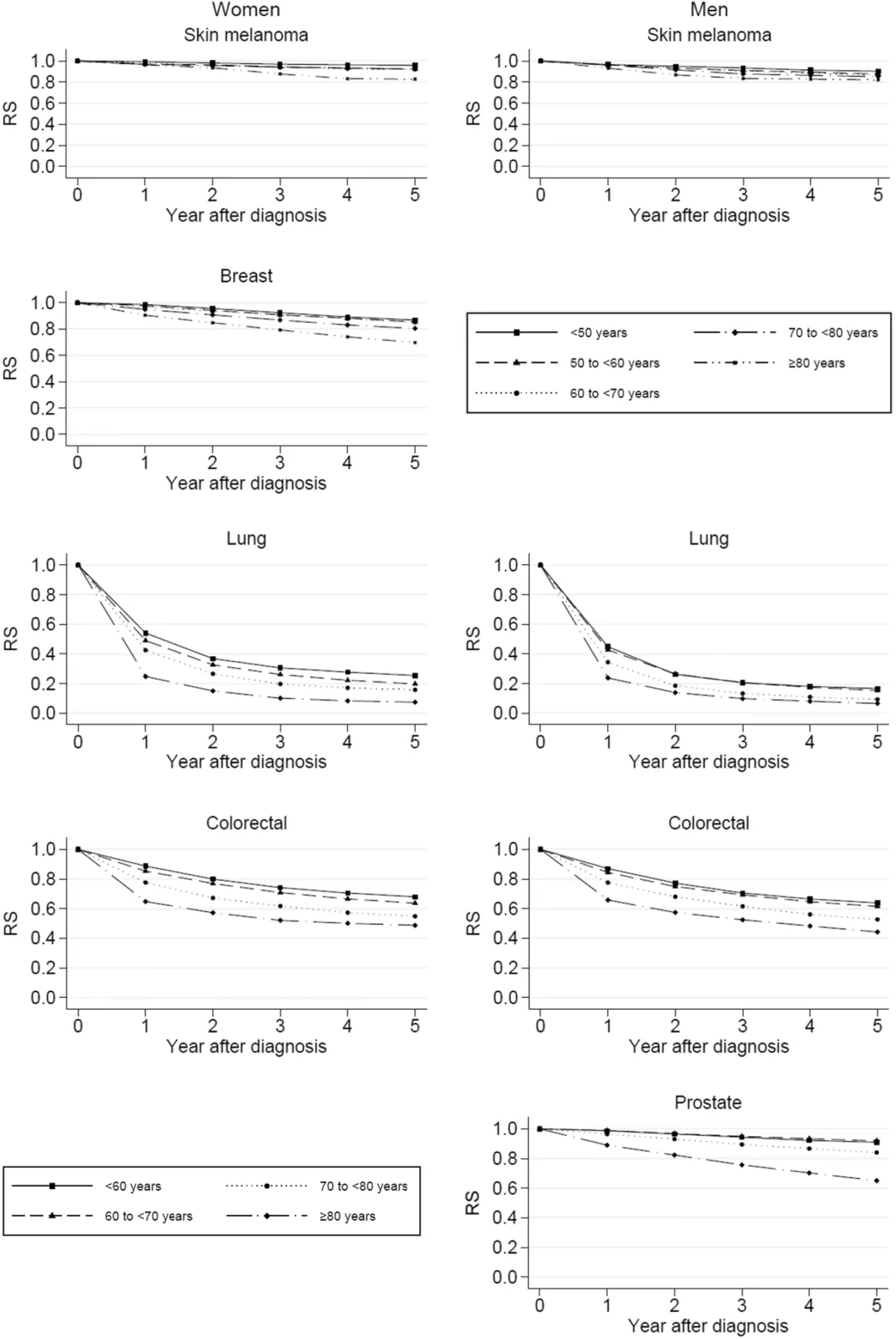
Relative survival (RS) up to 5 years after diagnosis for each cancer type and sex, by age group. Canton of Zurich, Switzerland, 1980–2015. Cohort analyses. Age groups for skin melanoma and breast cancer: <50 years, 50 to <60 years, 60 to <70 years, 70 to <80 years, ≥80 years; age groups for colorectal, lung and prostate cancer: <60 years, 60 to <70 years, 70 to <80 years, ≥80 years.

Figure 3 and Table S2 present the 5‐year relative survival estimates by cancer type, sex, and age group at incidence. In general, the 5‐year relative survival was similar in all the age groups up to 69 years and clearly lower in the age group ≥80 years. The estimates of the age group 70–79 years were mostly in between the younger age groups and those aged 80+ years. For example, the 5‐year relative survival was around 0.85 for women with breast cancer up to the age of 69 years but only 0.70 (0.67, 0.73) for women aged 80 years or older at the time of cancer diagnosis.

## 4 DISCUSSION

We observed increasing trends in age‐standardized 5‐year relative survival rates for breast, prostate, lung, colorectal cancer, and skin melanoma in the time periods between 1980 and 1989, and 2010 and 2015 in men and women living in the canton of Zurich in Switzerland. Independent of the period and the cancer type, survival tended to be slightly higher in women compared to men.

Furthermore, the age‐standardized 5‐year relative survival was lowest in patients with stage IV tumors for all cancer types and tended to decrease with increasing stage. For breast, prostate, colorectal cancer and skin melanoma, relative survival was relatively high up to stage III but dropped immensely for stage IV tumors. Only for lung cancer, the survival was gradually decreasing with increasing stage.

Regarding stratification by age group, lower survival was mainly observed in the oldest age group (80+ years) while it was mostly comparable in younger age groups.

### 4.1 Changes over time

In Switzerland, between 1995 and 1999, and 2005 and 2009, an increase in 5‐year relative survival has also been reported for breast cancer patients,^17^ for prostate cancer patients,^18^ for colon cancer patients,^19^ and for lung cancer patients.^20^ Increasing survival trends over time have also been observed in other countries and regions.^8^, ^21^, ^22^

For example, the International Cancer Benchmarking Partnership reported an increase in 5‐year relative survival for breast, colorectal and lung cancer in several countries between 1995 and 1999, and 2005 and 2007,^22^ comparable to the increase that we found in Zurich. CONCORD‐3 reported trends in 5‐year net survival between the time periods 2000–2004 and 2010–2014 for 18 cancer sites in 71 countries.^9^ This large international study reported increases in 5‐year net survival for breast, colorectal, lung cancer and skin melanoma for Switzerland (10 regional registries) that are comparable to our results.^9^ Another international study on cancer survival, EUROCARE‐5, reported trends in 5‐year relative cancer survival between the time periods 1999–2001 and 2005–2007.^8^ The reported estimates for Central Europe (which includes Switzerland) were comparable to our results for all cancer types.

Generally, relative survival increased for these common cancer types in many countries over the past decades,^4^, ^8^, ^9^, ^22^, ^23^ which was replicated with our data from the canton of Zurich. It has further been observed that substantial improvements have been achieved in Southern and Eastern European regions, where the survival rates have traditionally been lagging behind compared to the rest of Europe.^4^, ^7^

### 4.2 Cancer survival by stage

Similar to our study, other studies showed that cancer survival is quite high (sometimes even higher than 100%) if diagnosed at an early stage, for example for breast cancer in an Estonian (2005–2009),^24^ a German (2004–2008),^25^ and another Swiss study (2003–2012).^26^

For skin melanoma, studies in Denmark (2004–2008), Germany (2002–2011),^27^ and the Netherlands (2015)^28^ reported comparable results.

Studies from Germany and the USA^29^ as well as another Swiss study including data from several cancer registries/regions^30^ confirm our data for prostate cancer, where 5‐year relative survival was similarly high for patients with stage I, II and III tumors but clearly lower in patients with stage IV (distant) tumors. The reason is probably that for prostate cancer, lymphogenic metastases are only associated with stage IV, while for other cancer types, these are also associated with stages II and III while stage IV is mainly associated with distant metastases.

A study from the USA based on CONCORD‐2 data^31^ and also an international study including seven high‐income countries^32^ reported comparable results regarding 5‐year net survival for colon cancer to what we found for colorectal cancer.

Comparable results to our study were also reported for lung cancer in a study from the USA based on CONCORD‐2 data in 2004–2009,^33^ as well as in an Estonian study in 2010–2016.^34^

### 4.3 Cancer survival by age group

In general, lower survival for elderly patients is reported compared to younger patients for several cancer sites.^35^ Furthermore, a EUROCARE analysis showed that the survival gap between elderly and middle‐aged cancer patients is widening in Europe.^35^

The international study cited above reported similar survival estimates for colorectal cancer by age group in 2010–2014 with higher estimates up to 79 years and lower estimates for patients aged 80 years and older in most countries.^32^ Similar patterns were observed in Spain in 1995–1999^36^ and in 2002–2013^23^ and also in the SUDCAN study in six European Latin countries in 2004.^37^, ^38^

Regarding breast cancer survival by age group, comparable results to our study were reported in Estonia in 2005–2009,^24^ in another Swiss study in 2003–2012,^26^ and also in a German study in 2004–2008.^25^

Regarding prostate cancer survival by age group, similar estimates were reported in another Swiss study in 2000–2013,^30^ in Spain in 1995–1999^36^ and in 2002–2013.^23^

According to EUROCARE‐5, the patterns for 5‐year relative survival in relation to skin melanoma was comparable in European populations,^39^ and also according to a Spanish study in 2002–2013.^23^

A more gradual decline by age in 5‐year survival of lung cancer was reported in other studies such as the SUDCAN study in six European Latin countries in 2004^40^ and a Spanish study in 1995–1999.^36^

### 4.4 Reasons influencing cancer survival

The survival improvement observed over time may be due to different reasons. An obvious factor may be the uptake of screening in a population, leading to a stage‐shift with more lower‐staged tumors being diagnosed and thus improved survival. In the Canton of Zurich, there are no organized screening programs. Opportunistic screening exists for cervical, breast, prostate, and colorectal cancer. However, this information is not assessed in our database and can therefore not be associated with the survival data. Data for Switzerland show that the proportion of men aged 50 years and older being screened for prostate cancer within the last 2 years increased from 33% in 1992 to 42% in 2012.^41^ A big difference was observed in mammography attendance during the last 2 years for women aged 50–69 years between the French speaking part of Switzerland with organized screening programs (78%) and the German speaking part with only opportunistic screening (35%) in 2012.^42^ Colorectal cancer screening (fecal occult blood testing or colonscopy) during the last 2 years in individuals aged 50–75 years increased in the German‐speaking part from 34% in 2007 to 48% in 2017.^43^

Regarding the potential influence of stage‐shift, an earlier publication from the Cancer Registry Zurich has reported the changes in age‐standardized incidence rates by stage.^3^ There were some increases in lower‐staged tumors, for example in prostate cancer, however, a simultaneous increase in stage IV tumors was observed for example in colorectal cancer.^3^ Therefore, opportunistic screening in the Canton of Zurich does not seem to be the driving force for improvements in survival. However, improved diagnostics not related to screening and other technological advances that enable better diagnosis and staging such as PET‐CT may play a role. Furthermore, treatments have become more effective due to technological advancements such as improvements in surgical techniques, better tailoring of treatment options according to the patients characteristics including age, stage, comorbidities etc., and completely new technologies such as immune therapy, for example in the treatment of lung cancer. Moreover, the adherence to national and international treatment guidelines may have improved over time, which could also influence survival.

Finally, a raised awareness around cancer, such as breast self‐examination in women, may also lead to earlier detection and therefore better survival.

Similar to other studies, we have observed a poorer survival in patients aged 80 years and older. One reason may be the higher prevalence of comorbidities in this age group compared to younger patients, likely leading to less diagnostics and less treatment but more palliative care. Furthermore, younger patients may tolerate more aggressive treatments, and the take‐up of treatments in older patients may be lower, which leads to less treatment success. The considerably lower survival in stage IV patients is not surprising due to the metastatic disease, but emphasizes the need for early detection in order to further improve survival of cancer patients.

The strengths of this study are the long follow‐up period that allowed for observing trends over 35 years in the canton of Zurich, which along with the good data quality of the cancer registry data^11^ and high completeness provides robust results. In our study, we additionally provide estimates stratified by stage and age group, to highlight important differences that could inform future treatment‐related decisions in these groups. However, the study has also some limitations. Cancer registration only became mandatory in Switzerland in 2020, therefore we only included complete data for the canton of Zurich, since so few regions in Switzerland began cancer registration from such a long time ago. Furthermore, due to the large amount of missing data for stage in earlier years, we only included data from 2003 onwards in the stage‐stratified analyses.

### 4.5 Conclusions

This study describes, for the first time, trends in 5‐year relative survival for the most common cancer types over a long period of time (1980–2015) in the largest Swiss Canton (Zurich). We found that 5‐year relative survival rates increased for breast, prostate, lung, colorectal cancer and skin melanoma between 1980 and 2015 and was generally slightly higher in women compared to men. Reasons for the observed survival improvements likely include technological advancements in diagnostic methods and treatments, as well as treatments that are tailored to the patients' characteristics and new treatments such as immune therapy.

Survival was generally high for stage I tumors and low for stage IV tumors, while survival for stage II and III tumor patients was either reasonably high (breast, prostate, colorectal cancer, skin melanoma) or intermediate (lung cancer). This emphasizes the importance for early detection of tumors with the help of (organized) screening programs as well as new and improved diagnostic methods.

For all cancer types, survival was comparable for all age groups up to 80 years but remarkably lower in the oldest age group (80+ years). This is likely due to the higher prevalence of comorbidities in this age group, which may lead to less diagnostics and less treatment.

Finally, the increasing trends in survival may also reflect raised awareness around cancer and therefore earlier detection and treatment.

## AUTHOR CONTRIBUTIONS

**Miriam Wanner:** Conceptualization (equal); formal analysis (lead); methodology (equal); writing – original draft (lead). **Maria‐Eleni Syleouni:** Formal analysis (supporting); methodology (equal); writing – review and editing (equal). **Nena Karavasiloglou:** Formal analysis (supporting); methodology (equal); writing – review and editing (equal). **Manuela Limam:** Data curation (equal); writing – review and editing (equal). **Esther Bastiaannet:** Formal analysis (supporting); methodology (equal); writing – review and editing (equal). **Dimitri Korol:** Conceptualization (equal); data curation (equal); methodology (equal); writing – review and editing (equal). **Sabine Rohrmann:** Conceptualization (equal); methodology (equal); supervision (lead); writing – review and editing (equal).

## FUNDING INFORMATION

The authors conducted this study within their regular working contracts; no additional funding was obtained for the study.

## CONFLICT OF INTEREST STATEMENT

The authors have no conflict of interest.

## DISCLAIMER

At the time of publication, Dr. Karavasiloglou is an EFSA staff member. At the time of preparation of this work, Dr. Karavasiloglou was affiliated with the University of Zurich. Where authors are identified as personnel of the European Food Safety Authority, the authors alone are responsible for the views expressed in this article and they do not necessarily represent the decisions, policy or views of the European Food Safety Authority.

## Supporting information


Table S1.

Table S2.

## Data Availability

Due to legal reasons, the cancer registry data is not publicly available. Further details are available from the corresponding authors upon request.
