# Supplementary material for: Time‐trends and age and stage differences in 5‐year relative survival for common cancer types by sex in the canton of Zurich, Switzerland
Source: Cancer Med. 2023 Aug 1;12(17):18165–75. doi: 10.1002/cam4.6392 (PMC10524019; doi:10.1002/cam4.6392)
Supplement: Supplementary file 1 — Table S1. [file CAM4-12-18165-s002.pdf]

Supplementary Table 1. Age-standardized 5-year relative survival (ASR) and 95% confidence intervals (95% CI) for each cancer type and stage at diagnosis by sex. Canton of Zurich, Switzerland, 2003-2015. Cohort analyses.

Stage at diagnosis	5-year ARS	95% CI	5-year ARS	95% CI
	**Men**		**Women**	
**Breast cancer**				
Overall			**0.88**	(0.87; 0.89)
Stage I			**1.008**	*not calc.*[1)]
Stage II			**0.94**	(0.93; 0.95)
Stage III			**0.75**	(0.73; 0.78)
Stage IV			**0.29**	(0.25; 0.32)
Stage missing			**0.64**	(0.56; 0.71)
**Prostate cancer**				
Overall	**0.91**	(0.91; 0.92)		
Stage I	**0.98**	(0.95; 0.99)		
Stage II	**0.99**	(0.93; 1.00)		
Stage III	**1.009**	*not calc.*[1)]		
Stage IV	**0.58**	(0.54; 0.61)		
Stage missing	**0.89**	(0.86; 0.91)		
**Lung cancer**				
Overall	**0.19**	(0.17; 0.20)	**0.24**	(0.22; 0.25)
Stage I	**0.69**	(0.64; 0.74)	**0.76**	(0.70; 0.80)
Stage II	**0.50**	(0.43; 0.57)	**0.52**	(0.43; 0.61)
Stage III	**0.20**	(0.17; 0.24)	**0.26**	(0.22; 0.30)
Stage IV	**0.04**	(0.03; 0.05)	**0.07**	(0.05; 0.08)
Stage missing	**0.08**	(0.06; 0.10)	**0.13**	(0.10; 0.17)
**Colorectal cancer**				
Overall	**0.65**	(0.63; 0.66)	**0.68**	(0.66; 0.70)
Stage I	**0.97**	(0.91; 0.99)	**0.98**	(0.92; 0.99)
Stage II	**0.83**	(0.79; 0.86)	**0.88**	(0.85; 0.91)
Stage III	**0.67**	(0.64; 0.70)	**0.68**	(0.64; 0.71)
Stage IV	**0.13**	(0.11; 0.16)	**0.15**	(0.12; 0.18)
Stage missing	**0.59**	(0.54; 0.64)	**0.70**	(0.65; 0.74)
**Skin melanoma**				
Overall	**0.92**	(0.91; 0.94)	**0.96**	(0.94; 0.97)
Stage I	**1.011**	*not calc.*[1)]	**1.013**	*not calc.*[1)]
Stage II	**0.84**	(0.73; 0.91)	**0.88**	(0.80; 0.93)
Stage III	**0.66**	(0.56,;0.75)	**0.63**	(0.50; 0.73)
Stage IV	**0.15**	(0.07; 0.25)	**0.28**	(0.14; 0.44)
Stage missing	**0.92**	(0.90; 0.94)	**0.96**	(0.93; 0.97)

[1)] CI of estimates >1 are not calculated (see https://pauldickman.com/software/strs/history/history/, 20130329 Version 1.3.8)
