# Supplementary material for: Time‐trends and age and stage differences in 5‐year relative survival for common cancer types by sex in the canton of Zurich, Switzerland
Source: Cancer Med. 2023 Aug 1;12(17):18165–75. doi: 10.1002/cam4.6392 (PMC10524019; doi:10.1002/cam4.6392)
Supplement: Supplementary file 2 — Table S2. [file CAM4-12-18165-s001.pdf]

**Supplementary Table 2. 5-year relative survival estimates (RS) and 95% confidence intervals (95% CI) for each cancer type and age at cancer diagnosis by sex. Canton of Zurich, Switzerland, 1980-2015. Cohort analyses.**

| Age at diagnosis | 5-year RS | 95% CI | 5-year RS | 95% CI |
|---|---|---|---|---|
| | **Men** | | **Women** | |
| **Breast cancer** | | | | |
| Overall | | | **0.83** | (0.83; 0.84) |
| <50 years | | | **0.87** | (0.86; 0.88) |
| 50-59 years | | | **0.85** | (0.84; 0.86) |
| 60-69 years | | | **0.85** | (0.84; 0.86) |
| 70-79 years | | | **0.81** | (0.79; 0.82) |
| ≥80 years | | | **0.70** | (0.67; 0.73) |
| **Prostate cancer** | | | | |
| Overall | **0.85** | (0.85; 0.86) | | |
| <60 years | **0.91** | (0.90; 0.92) | | |
| 60-69 years | **0.92** | (0.91; 0.93) | | |
| 70-79 years | **0.84** | (0.83; 0.85) | | |
| ≥80 years | **0.65** | (0.62; 0.68) | | |
| **Lung cancer** | | | | |
| Overall | **0.13** | (0.12; 0.14) | **0.19** | (0.18; 0.20) |
| <60 years | **0.17** | (0.15; 0.18) | **0.25** | (0.23; 0.28) |
| 60-69 years | **0.15** | (0.14; 0.16) | **0.20** | (0.18; 0.22) |
| 70-79 years | **0.09** | (0.08; 0.10) | **0.16** | (0.14; 0.18) |
| ≥80 years | **0.07** | (0.05; 0.08) | **0.07** | (0.05; 0.10) |
| **Colorectal cancer** | | | | |
| Overall | **0.57** | (0.55; 0.58) | **0.58** | (0.57; 0.59) |
| <60 years | **0.64** | (0.62; 0.66) | **0.68** | (0.66; 0.70) |
| 60-69 years | **0.62** | (0.59; 0.64) | **0.64** | (0.61; 0.66) |
| 70-79 years | **0.53** | (0.51; 0.55) | **0.55** | (0.53; 0.57) |
| ≥80 years | **0.44** | (0.41; 0.48) | **0.49** | (0.46; 0.52) |
| **Skin melanoma** | | | | |
| Overall | **0.87** | (0.86; 0.88) | **0.93** | (0.92; 0.94) |
| <50 years | **0.90** | (0.88; 0.92) | **0.96** | (0.95; 0.97) |
| 50-59 years | **0.88** | (0.85; 0.90) | **0.92** | (0.90; 0.94) |
| 60-69 years | **0.87** | (0.84; 0.89) | **0.92** | (0.90; 0.94) |
| 70-79 years | **0.85** | (0.82; 0.88) | **0.92** | (0.89; 0.95) |
| ≥80 years | **0.82** | (0.74; 0.90) | **0.83** | (0.76; 0.89) |
